# Artificial Intelligence in Imaging for Personalized Management of Coronary Artery Disease

**DOI:** 10.3390/jcm14020462

**Published:** 2025-01-13

**Authors:** Adrian Bednarek, Karolina Gumiężna, Piotr Baruś, Janusz Kochman, Mariusz Tomaniak

**Affiliations:** First Department of Cardiology, Medical University of Warsaw, Banacha 1a, 02-097 Warsaw, Poland

**Keywords:** artificial intelligence, personalized therapy, deep learning, machine learning

## Abstract

The precision of imaging and the number of other risk-assessing and diagnostic methods are constantly growing, allowing for the uptake of additional strategies for individualized therapies. Personalized medicine has the potential to deliver more adequate treatment, resulting in better clinical outcomes, based on each patient’s vulnerability or genetic makeup. In addition to increased efficiency, costs related to this type of procedure can be significantly lower. Useful assistance in designing individual therapies may be assured by the adoption of artificial intelligence (AI). Recent years have brought essential developments in deep and machine learning techniques. Advances in technologies such as convolutional neural networks (CNNs) have enabled automatic analyses of images, numerical data, and video data, providing high efficiency in the creation of prediction models. The number of AI applications in medicine is constantly growing, and the effectiveness of these techniques has been demonstrated in coronary computed tomography angiography (CCTA), optical coherence tomography (OCT), and many others. Moreover, AI models may be useful in direct therapy optimization for patients with coronary artery disease (CAD), who are burdened with high risk. The combination of well-trained AI with the design of individual treatment pathways can lead to improvements in health care. However, existing limitations, such as non-adapted guidelines or the lack of randomized clinical trials to evaluate AI’s true accuracy, may contribute to delays in introducing automatic methods into practical use. This review critically appraises the developed tools that are potentially useful for clinicians in guiding personalized patient management, as well as current trials in this field.

## 1. Introduction

The primary interest in the connection between AI and medicine was presented in the 1950s [1]. AI enabled the discovery of specific patterns in large amounts of data, the analysis of which is often beyond the scope of research teams, contributing to the development of algorithms achieving high diagnostic accuracy in a relatively short time. Recent years have been especially abundant with publications reporting the implementation of AI-based models. New technical solutions have accelerated the development of algorithms based on machine learning (ML) [2,3,4], contributing to the diagnosis of many diseases and prediction of their subsequent outcomes, improvement in radiographic image quality, the establishment of new risk scales, and even treatment guidance [5,6,7,8,9].

**Figure 1:**
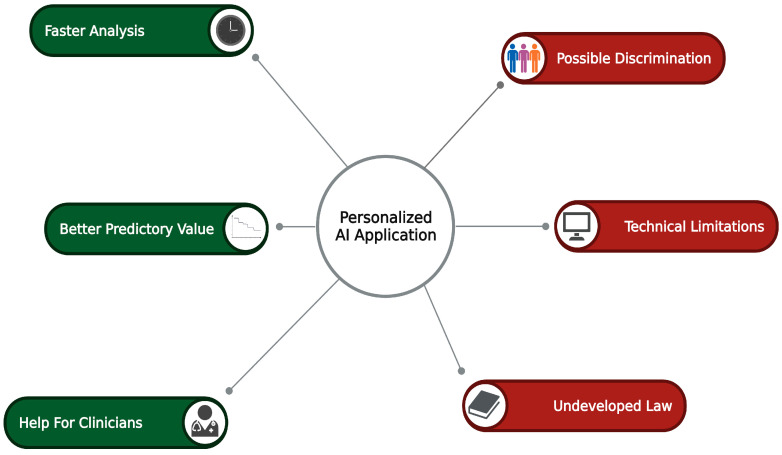
Benefits and pitfalls of AI usage in personalized medicine AI—artificial intelligence. Created with Biorender.com.

“Personalized medicine”, also known as “precision medicine”, has many definitions; nevertheless, this conception focuses on maximizing treatment efficiency through therapy individualization. This term first appeared in an article published in 1999 [10]. Twenty-five years later, this concept is increasingly considered in the context of treatment recommendations. In practice, it is not currently widely used due to its high introduction cost and lack of an appropriate technical background (Figure 1) [11]. Coronary artery disease (CAD), which includes both acute states requiring rapid treatment decisions and chronic states requiring very precise and tailored management of the disease, may particularly benefit from an individualized approach due to its high prevalence and different patient characteristics [12,13,14].

An increase in personalized approach availability and higher efficiency achievement can be provided by the integration of AI methods. Automatic assistance in therapy guidance and outcome estimation will allow for the adoption of less time-consuming and safer solutions that may be included in future recommendations on a larger scale. We present a review of potentially useful algorithms that help in disease assessment and optimize invasive or pharmacological treatments, highlighting their possible influence on the progress of personalized approaches.

## 2. Evaluation of Coronary Anatomy and Stenosis

The decision process for the choice of percutaneous coronary intervention (PCI) starts with coronary anatomy evaluation and visual stenosis assessment. Current guidelines recommend that noninvasive imaging (such as coronary computed tomography angiography (CCTA), cardiac magnetic resonance (CMR), and single-photon emission computed tomography (SPECT)) should be the first choice for chronic coronary syndrome assessment in patients with low, moderate, and high risk factor-weighted clinical likelihood to minimize procedural risk [12,14]. CCTA is a widely used method for the primary evaluation of vessel lumen narrowing. It should be highlighted that CCTA carries some limitations—it may overestimate the degree of stenosis, which lowers its specificity [14]. Nevertheless, its high negative predictive value is an advantage for the non-invasive exclusion of CAD [5].

To standardize the stenosis assessment, the CAD Reporting and Data System (CAD-RADS) scale, based on the degree of stenosis, was developed to distinguish patients needing the implementation of additional diagnostic methods to evaluate the significance of the lesion. A model based on a convolutional neural network (CNN) allowed for differentiation between CAD-RADS 0–2 and CAD-RADS 3–5 with a sensitivity of 82% and specificity of 58%, five times quicker compared to on-site physician readers (530.5 ± 179.1 vs. 104.3 ± 1.4 s, *p* = 0.01) [15]. This approach allows automatic identification of patients with stenosis ≥50% who require further functional or invasive evaluation. In turn, the CLARIFY trial showed that AI had an excellent agreement in identifying the potentially treatment-needing vessels (stenosis > 70%), being inconsistent with readers in only one case either per vessel or per patient [16]. The substudy of the CREDENCE trial also showed a high AUC (0.92) of AI-derived quantitative coronary angiography (QCA) compared to the manual approach in detecting 70% stenosis [17]. Despite the false positive results in 62 cases compared to the manual approach, 66.1% of patients in this subgroup had FFR < 0.8, which may help in revealing patients with a high probability of significant functional disease even in the absence of severe stenosis [17]. The AI-QCA also performed significantly better than myocardial perfusion imaging in detecting obstructive CAD [18]. The CONSERVE trial, in which one group was referred for direct catheterization and another group first underwent coronary imaging and quantitative cardiac computed tomography with the commercially available software (Cleerly Labs, Cleerly, Inc., New York, NY, USA), revealed that the CCTA strategy can reduce the number of unnecessary coronary angiographies and may be more financially profitable without changes in 1-year major adverse cardiac event (MACE) risk [19]. The use of this FDA-approved software contributed to a significant increase in the prescription of preventive medication, as well as physician confidence and a reduction in the number of invasive procedures [20]. These data show that automatic, noninvasive analysis of stenosis profiles may greatly assist physicians and help in selecting optimal diagnostic pathways, balancing between invasive procedure risk and the probability of future ischemic events. Automatic detection and evaluation are needed for assessment standardization in strongly calcified lesions. Optimal stenosis estimation will lead to the reduction of invasive investigations, but adequate strategy implementation is limited without AI due to the long waiting time for expert assessment and the overestimation of stenosis by on-site readers in up to 40% of patients [21]. It should also be highlighted that current evidence supports the PCI planning based on physiological evaluation; thus, stenosis degree estimation with AI algorithms would rather serve as a rapid and consistent modality for accurate preliminary assessment and as a tool for the decision on further diagnostics, than for the treatment choice itself. Nevertheless, the implementation of automatic and accurate stenosis detection in CCTA will be significantly beneficial in reducing the number of coronary angiographies without subsequent PCI treatment, especially in smaller centers.

## 3. Functional Assessment for Ischemia

Additionally to the stenosis evaluation, current guidelines recommend the functional assessment of ischemia and myocardial viability in patients with chronic coronary syndromes, depending on the risk factor-weighted clinical likelihood [14]. The suggested methods especially include CMR, positron emission tomography (PET), and SPECT. Myocardial ischemia in CMR can be assessed automatically with the AI tool with high accuracy (86.39%), sensitivity (90%), and AUC = 0.93 [22]. However, this solution was based on a limited number of scans and needs further clinical evaluation. Changes in the myocardium surface can also deliver practical information, so Chen et al. proposed a deep learning (DL)-based segmentation that can distinguish between scar tissue and normal myocardium, deriving superior results compared to commonly used Gaussian mixture models [23]. It turned out that the dense scar mass revealed after late gadolinium enhancement (LGE) derived by the model may be a predictive factor of MACE at a cut-off value of 20.3 g with HR = 2.35 (95% CI 1.33–4.15, *p* = 0.003). However, in multivariate analysis, the guideline criterion for implantable cardioverter defibrillator was the only significant variable, whereas dense scar mass predictive value achieved *p* = 0.05 [24].

Frequently, acute coronary syndrome may present with the absence of obstructive coronary stenosis, and instead, coronary microvascular dysfunction can constitute its cause [25]. Myocardial blood flow (MBF) may be used for the evaluation of microvessel functionality and can be precisely estimated with PET. PET’s capacity to diagnose abnormal perfusion allows for its use as an independent estimator of MACE [26,27]. An ML model reached AUC = 0.71 in the detection of MACE-increased risk using myocardial perfusion reserve [28]. Furthermore, a similar effect was obtained regarding myocardial ischemia (AUC = 0.72) [28]. This approach may identify patients who will benefit from applying and intensifying treatment of microvascular dysfunction, which is currently underdiagnosed and insufficiently managed due to the requirement for additional diagnostic steps and the not fully understood pathomechanisms leading to its presence.

There is consensus that the decision for PCI should be based on physiological assessment, such as the fractional flow reserve (FFR) index, rather than visual estimation alone, due to the high disconcordance of these approaches and the proven superiority of functional assessment [29]. AI may provide a noninvasive, onsite, direct estimation of FFR value from CCTA [30], which is currently limited due to the high computational power required for computational fluid dynamics (CFD) simulation and high cost. The FFR index based on AI may influence the treatment pathway by reducing the number of coronary angiographies and revascularization procedures compared to an a priori coronarography strategy [31]. Moreover, in cases of heavy calcifications, which could interfere with FFR_CT_ measurements, FFR can be derived with AI use from a combined approach with CCTA and angiography (FFR_CT-angio_) [32]. FFR_CT-angio_ had a strong correlation with invasive FFR (r = 0.81, *p* < 0.001) and achieved 100% sensitivity and 88.8% specificity in identifying ischemic lesions [32]. Automatization and improvement of FFR measurements and stenosis degree from CCTA will constitute a tool for more accurate disease assessment and better therapy adjustment without the need for performing invasive coronary angiography.

In addition, FFR_CT_ can be used not only for PCI guidance but also for non-invasive coronary assessment before non-cardiological procedures. In patients before liver transplantation, the specificity and negative predictive value in evaluating CAD were 90% (82–98%) and 91% (84–99%), respectively [33]. CT scans are often routinely performed for patients qualified for surgical treatment. Therefore, FFR_CT_ may serve as a useful tool for faster and safer coronary assessment before these procedures, especially considering the current guidelines, which suggest minimizing the use of additional diagnostic methods [34]. The adoption of AI might lead to wider availability of noninvasive, functional assessment of CAD, which can reduce invasive angiography by even 54% without a significant reduction in the number of PCI procedures [31].

Recent evidence also supports the concept of shear stress’s influence on vulnerability and future MACE [35]. The connection of high-risk plaque features and adverse hemodynamic parameters, including shear stress, can accurately predict subsequent ACS and may help in identifying patients requiring intensified personalized care [35]. Apart from the CFD solutions based on angiograms, accurate radial wall strain (RWS) assessment can be performed by AI from optical coherence tomography (OCT). RWS incorporation may provide insight into biomechanical forces influencing plaque vulnerability, especially due to the correlation with high-risk features such as thin-cap fibroatheroma (TCFA) and lipid-to-cap ratio [36].

## 4. Morphological Assessment of the Lesion

Current evidence suggests that the morphological evaluation of atherosclerotic plaque may reveal factors influencing the risk of future ischemic events [37,38]. The appearance of the plaque alone is not yet the determinant of increased risk of a cardiovascular incident; thus, the term “vulnerable plaque” was created to describe those prone to rupture [39]. These findings highlight that therapeutic strategy may be dependent not only on physiological features but also on the morphological profile of the plaque. Recent evidence shows the potential beneficial effect of vulnerable plaque stenting even in non-flow limiting lesions, supporting the implementation of a tailored strategy based on several individual features [40]. The noninvasive assessment of morphological plaque profiles in CCTA became possible due to AI. The REVEALPLAQUE trial showed the high accuracy of AI-QCPA software developed by HeartFlow in plaque morphology quantification compared with intravascular ultrasound (IVUS) as a gold standard [41]. Moreover, the DECODE study revealed that AI-QCPA-based analysis led to a change in clinical care in two-thirds of patients [42]. Plaque morphology evaluation mainly contributed to the intensification of lipid-lowering therapy, which is a promising concept in terms of personalized selection of pharmacological strategy. It may play significant role in treatment guidance in high-risk patients, especially considering the insufficiency of current therapy schemes in achieving lipid goals [43,44,45]. DL has enabled more sophisticated analyses regarding the characterization of the plaque type, volume, burden, and vessel stenosis with excellent or good agreement (intraclass correlation coefficient from 0.801 to 0.992), five times faster than human readers (5.65 ± 1.87 s vs. 25.66 ± 6.79 min) [46]. Furthermore, parameters derived by AI had significant prognostic value—patients with total plaque volume ≥ 238.5 mm^3^ had seven times higher risk of myocardial infarction (MI), and patients with stenosis ≥ 50% had three times higher risk of MI [46]. It is also possible to differentiate between plaque types using ML; however, calcified plaques are sometimes excluded, limiting this method’s usefulness [47]. AI-quantitative CT was also able to detect high-risk CT features, such as low-density non-calcified plaque ≥ 2.3 mm^3^ and a remodeling index > 1.1, which may allow a fast-track approach for patient risk assessment and further diagnostic evaluation before the time-consuming, extensive description of images by radiologists.

The current gold standard of plaque morphology assessment is intravascular imaging, which allows for precise intraluminal evaluation with IVUS or optical coherence tomography (OCT). The urgent need for AI use in IVUS guidance is due to existing inter-institute variability [48], which may cause irregularities in personalized therapeutic decisions based on the luminal measurements and calcium profiles. Like other imaging methods, DL in IVUS is mainly adopted in the correction of the resolution and segmentation [49,50], facilitating this arduous process. OCT-AI techniques empower the automatic detection and characterization of plaques in coronary arteries [8,51], being especially useful in evaluating the presence of TCFA, which is associated with an increased MACE risk [52]. Algorithms may also provide great assistance in identifying and classifying calcifications, aiding in stenting optimization and tailored therapeutic choices [53,54,55]. Automatic marking of plaque erosion, which is responsible for 25–50% of MI, has become possible with AI assistance, yielding a high negative predictive value at both frame and lesion levels [56]. Accurate detection of this pathology is crucial because of current evidence showing that conservative treatment can be applied in those cases instead of stenting [57]. On the other hand, the plaque profile may be also an indication for the use of different PCI modalities. A calcium angle greater than 180 degrees may cause procedural difficulties and is associated with a higher rate of stent under-expansion, contributing to a higher rate of restenosis [55,58]. An algorithm derived an accuracy of 0.98 for detecting calcifications present in more than two quadrants, which may speed up the decision on the choice of therapeutic method [59]. AI features detecting calcium angle and thickness are now commercially available (Ultreon™ 2.0 Software, Abbott, Chicago, IL, USA). The assessment of the calcium angle may serve as great assistance in the treatment choice with the use of calcium modification techniques such as rotational atherectomy or intravascular lithotripsy, which improve procedural results [60].

Not only the plaque type assessment but also intraluminal measurements may be useful in PCI guidance. It was proven that non-culprit vessels with a minimal lumen area (MLA) under 4 mm^2^ are associated with a higher risk for MACE. The algorithm assessed this cut-off point in patients with stable angina pectoris and yielded an accuracy of 0.97 [59]. In turn, the model predicting the formation of or remaining high-risk plaques such as TCFA, plaque burden ≥ 70%, or MLA < 4 mm^2^ in patients with lipid-lowering therapy achieved a G-mean values of 78.6%, 81.2%, and 84.5%, respectively, for each feature at 1 year [61]. Assessing the risk of vulnerable plaque presence in the future and introducing it into schemes for more precise lipid-lowering therapy choices and adjusting their goals should be tested to evaluate the real clinical utility of tailored strategies based on morphological features [62]. Tools such as OCTPlus software are currently adopted for prospective trials regarding plaque compositions [63]. Another algorithm named OCTOPUS is also widely used in trials [64]. A detailed review of plaque-type characterization by AI was published recently [65]. Current technical advancements have enabled the combination of different modalities to derive precise information about the luminal environment and plaque vulnerability. A feasibility study on combining OCT and IVUS imaging with wall shear stress measurements was conducted, but only two arteries were included in the analysis, making it difficult to derive conclusions [66]. Nevertheless, this approach shows the possibility of connecting morphological, physiological, and biomechanical parameters, which should be the most desired way of assessing a patient’s risk.

In current clinical practice, several modalities for PCI optimization are used; however, despite the proven efficacy of stents, cases of their under-expansion still occur. Novel methods may help in determining the risk of insufficient stent expansion, which may help in PCI optimization. An AI-based solution enabled the estimation of predicted stent area (r = 0.802) before stenting, using IVUS pullback [67]. Furthermore, stent deployment calcification scores were defined as comparable to those obtained by human experts in four out of five cases [54]. A similar approach to predicting stent under-expansion was also applied regarding OCT [6]. The clinical use of these types of predictors will aid in estimating the risk of in-stent restenosis or stent thrombosis [68] and will allow more careful intra-procedural optimization of the management of CAD by IVUS and OCT-driven automatic devices. The automatic estimation of optimal stent choice and positioning may be revolutionary in optimizing treatment, not only based on the stenosis profile but also in connection with flow-related indices such as post-PCI FFR values and shear stress estimations. This could enable the adequate tailoring of the procedure based on the morphological, biomechanical, and physiological profile, reducing the probability of future ischemic events [13,69].

## 5. Prediction of Clinical Outcomes

ML development has enabled not only the establishment of high-risk features but also the prediction of adverse events from combined data. The CONFIRM study demonstrated the effectiveness of combining CCTA findings and clinical features using ML, yielding a risk score classification with the highest AUC (0.79) in predicting 5-year all-cause mortality. This approach outperformed traditional methods, including the segment stenosis score, segment involvement score, modified Duke index, number of segments with non-calcified, mixed or calcified plaques, age, sex, standard cardiovascular risk factors, and the Framingham risk score (*p* < 0.001 for all) [70]. This evidence suggests that while commonly described risk factors are useful for predicting events, their integration with AI-based methods will enable the most optimal risk classification.

The introduction of the fat attenuation index (FAI) allows for better and more tailored risk prediction using CCTA images. FAI measures differences in water and lipid content, typically present due to coronary inflammation from plaque formation or destabilization [71]. Current data suggest the association between the fat radiomic profile and MACE risk (HR 1.12 *p* < 0.001 per 0.01 increment) and the presence of inflammation-based FAI index differences near lesions [72]. A device estimating the risk of fatal cardiac events over 8 years was recently developed based on standardized FAI [73], improving risk discrimination and decision curve analysis compared to clinical and traditional factor-based models [73]. Some reports indicate that FAI, combined with other plaque feature analyses, may be more useful in stratifying high-risk patients than FFR_CT_ [74]. The integration of radiomic profiles, risk factors, serum lipids, and high-sensitivity C-reactive protein level has improved the discrimination of acute MI from stable or no CAD, achieving an AUC of 0.87, which was higher compared to models based only on clinical factors (AUC = 0.76) [75]. This capability may be valuable for differentiating patients with anginal symptoms but no significant coronary artery changes, potentially reducing the number of unnecessary invasive procedures. However, some reports are not very optimistic. The extension of the clinical model with AI-derived CCTA features resulted only in modest improvement of AUC, which may cool the enthusiasm a bit and lead to a discussion about the scope of benefits from implementing such comprehensive solutions [76].

**Figure 2:**
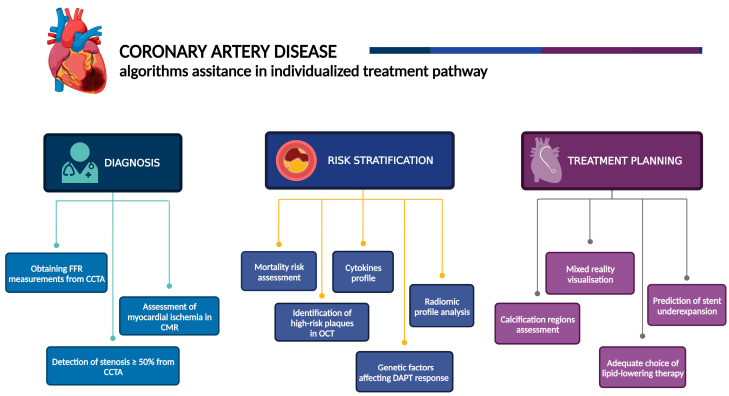
Algorithms’ assistance in the individualized treatment pathway. OCT—optical coherence tomography, CMR—cardiac magnetic resonance, CAD—coronary artery disease, CCTA—coronary computed tomography angiography, DAPT—dual antiplatelet therapy, FFR—fractional flow reserve. Created with Biorender.com.

MACE prediction can also be derived from ML analyses of SPECT myocardial perfusion imaging (MPI) and clinical variables. An algorithm achieved an AUC of 0.81 for 3-year MACE risk estimation, but the low annual MACE percentage (around 3%) in several similar studies may limit those methods’ validation processes [77,78]. An automatic predictor of CAD patient risk, based on fluorine-18 PET combined with CT-derived plaque quantification and a clinical characteristic model, yielded a C-statistic of 0.85, outperforming clinical risk scores and conventional coronary calcium CT analyses [79]. Additionally, an estimator of cardiac mortality risk based on 49 features (including clinical and SPECT-based data) achieved an AUC of 0.83 [80]. Echocardiography may also play a significant role in patient risk stratification. In a large study (812,278 echocardiographic videos), the algorithm was designed to predict the 1-year all-cause mortality [81]. The first test was based only on echocardiographic images, and the results were compared with the viewpoint of cardiology experts. The effectivity in prediction was significantly higher (AUC = 0.84) for a machine in comparison with an aggregated score of clinicians (AUC = 0.68) [81]. In the second step, to measure discrimination performance, a person with a higher mortality tendency from each of the 300 pairs was selected by AI and human specialists independently. Again, the algorithm turned out to be more effective (82% accuracy for the algorithm and 76%, 73%, 70%, and 66% for four cardiologists, respectively) [81], which allows for more vigilant care of the patient burdened with high predicted risk. The presented tools offer a perspective for more accurate and fully automatic evaluations of cardiac ischemia and its connection to MACE risk, which may potentially improve the adoption of personalized therapy or prevention approaches (Figure 2). More advanced technical details of AI applications in the cardiology field were described in several articles [2,3,9].

## 6. Challenges

Several additional, clinically oriented trials need to be conducted before the widespread adoption of AI solutions in daily clinical practice (Supplementary Table S1). Attention should be paid to achieving repeatability in more trials conducted on diverse groups, as most current models yield appropriate results in underpowered, retrospective studies that cannot be extrapolated for the entire population. Overfitting, when an algorithm performs well on the tested cohort but fails to generalize to the broader population, may constitute the main barrier to using automated solutions in clinical practice. The quality of the training process is crucial, but it is challenging to find a sufficiently diverse group. Moreover, numerous studies have revealed AI’s tendency for gender bias, age, and racial discrimination, which are linked to factors prevalent in some ethnic populations that were not included in training data [82,83].

One of the main challenges is also the insufficient technical background of the medical facilities. Even highly specialized hospitals usually do not have devices with adequate computational power, whereas online-based solutions may significantly burden the already strained network. Investments in AI solutions will require millions of dollars not only for hardware upgrades but also for personnel training, which may deepen the difference in the quality of healthcare between centers. Despite potential inclusion in the guidelines, advanced solutions may be underused due to the lack of experience and will of less technically acquainted staff; thus, appropriate training and awareness campaigns are needed. However, the wide prevalence of automated imaging techniques may encourage their widespread use and promotion of patient-first-imaging screening strategies in certain diseases, which may reduce overall costs. Nevertheless, the data are needed to evaluate their cost-efficacy and applicability in business models.

For patients, the main concern is the risk of sharing their sensitive data. A personalized approach involves storing much larger and more confidential information, which may be stolen. To prevent data leaks, special security measures must be considered. Before the wide application of AI, the legal and responsibility aspects should be reviewed as well. More general challenges are outlined in a recently published paper [84].

## 7. Conclusions

Due to the ability of algorithms to achieve similar or superior accuracy compared to clinicians, often in a shorter time, AI solutions will unquestionably aid the decision-making process in the management of CAD. The development of algorithms continuously contributes to the evolution of personalized medicine and will likely enable wider access to individualized treatment in the long term. This is due to the stratification of risk for each patient based not only on comorbidities but also on biomarkers, imaging patterns, and even genetic factors. At present, solutions aiding reliable invasive functional lesion assessment, such as FFR, appear to be the most advanced in terms of clinical translation and relevance for daily practice. However, before the wide implementation of AI, there are several issues that should be considered and reevaluated.

## Data Availability

This research did not generate any new data.

## Supplementary Materials

The following supporting information can be downloaded at: https://www.mdpi.com/article/10.3390/jcm14020462/s1, Table S1. Currently ongoing trials of AI-based tools for the management of coronary artery disease.

## Abbreviations

AI—artificial intelligence; PCI—percutaneous coronary intervention.

## References

[1] Ledley R.S., Lusted L.B. (1959). Reasoning foundations of medical diagnosis; symbolic logic, probability, and value theory aid our understanding of how physicians reason. Science.

[2] Maher G., Wilson N., Marsden A. (2019). Accelerating cardiovascular model building with convolutional neural networks. Med. Biol. Eng. Comput..

[3] Bizopoulos P., Koutsouris D. (2018). Deep Learning in Cardiology. IEEE Rev. Biomed. Eng..

[4] Abdulrazzaq M.M., Ramaha N.T.A., Hameed A.A., Salman M., Yon D.K., Fitriyani N.L., Syafrudin M., Lee S.W. (2024). Consequential Advancements of Self-Supervised Learning (SSL) in Deep Learning Contexts. Mathematics.

[5] White R.D., Erdal B.S., Demirer M., Gupta V., Bigelow M.T., Dikici E., Candemir S., Galizia M.S., Carpenter J.L., O’Donnell T.P. (2021). Artificial Intelligence to Assist in Exclusion of Coronary Atherosclerosis During CCTA Evaluation of Chest Pain in the Emergency Department: Preparing an Application for Real-world Use. J. Digit. Imaging.

[6] Gharaibeh Y., Lee J., Zimin V.N., Kolluru C., Dallan L.A.P., Pereira G.T.R., Vergara-Martel A., Kim J.N., Hoori A., Dong P. (2023). Prediction of stent under-expansion in calcified coronary arteries using machine learning on intravascular optical coherence tomography images. Sci. Rep..

[7] Mamas M.A., Roffi M., Frobert O., Chieffo A., Beneduce A., Matetic A., Tonino P.A.L., Paunovic D., Jacobs L., Debrus R. (2023). Predicting target lesion failure following percutaneous coronary intervention through machine learning risk assessment models. Eur. Heart J. Digit. Health.

[8] Abdolmanafi A., Duong L., Ibrahim R., Dahdah N. (2021). A deep learning-based model for characterization of atherosclerotic plaque in coronary arteries using optical coherence tomography images. Med. Phys..

[9] Naqvi R.A., Malik A.H., Kim H.S., Lee S. (2024). Transformative Noise Reduction: Leveraging a Transformer-Based Deep Network for Medical Image Denoising. Mathematics.

[10] Jorgensen J.T. (2009). New era of personalized medicine: A 10-year anniversary. Oncologist.

[11] Goetz L.H., Schork N.J. (2018). Personalized medicine: Motivation, challenges, and progress. Fertil. Steril..

[12] Byrne R.A., Rossello X., Coughlan J.J., Barbato E., Berry C., Chieffo A., Claeys M.J., Dan G.A., Dweck M.R., Galbraith M. (2023). 2023 ESC Guidelines for the management of acute coronary syndromes. Eur. Heart J..

[13] Tomaniak M., Katagiri Y., Modolo R., de Silva R., Khamis R.Y., Bourantas C.V., Torii R., Wentzel J.J., Gijsen F.J.H., van Soest G. (2020). Vulnerable plaques and patients: State-of-the-art. Eur. Heart J..

[14] Vrints C., Andreotti F., Koskinas K.C., Rossello X., Adamo M., Ainslie J., Banning A.P., Budaj A., Buechel R.R., Chiariello G.A. (2024). 2024 ESC Guidelines for the management of chronic coronary syndromes. Eur. Heart J..

[15] Muscogiuri G., Chiesa M., Trotta M., Gatti M., Palmisano V., Dell’Aversana S., Baessato F., Cavaliere A., Cicala G., Loffreno A. (2020). Performance of a deep learning algorithm for the evaluation of CAD-RADS classification with CCTA. Atherosclerosis.

[16] Choi A.D., Marques H., Kumar V., Griffin W.F., Rahban H., Karlsberg R.P., Zeman R.K., Katz R.J., Earls J.P. (2021). CT Evaluation by Artificial Intelligence for Atherosclerosis, Stenosis and Vascular Morphology (CLARIFY): A Multi-center, international study. J. Cardiovasc. Comput. Tomogr..

[17] Griffin W.F., Choi A.D., Riess J.S., Marques H., Chang H.J., Choi J.H., Doh J.H., Her A.Y., Koo B.K., Nam C.W. (2023). AI Evaluation of Stenosis on Coronary CTA, Comparison With Quantitative Coronary Angiography and Fractional Flow Reserve: A CREDENCE Trial Substudy. JACC Cardiovasc. Imaging.

[18] Lipkin I., Telluri A., Kim Y., Sidahmed A., Krepp J.M., Choi B.G., Jonas R., Marques H., Chang H.J., Choi J.H. (2022). Coronary CTA With AI-QCT Interpretation: Comparison With Myocardial Perfusion Imaging for Detection of Obstructive Stenosis Using Invasive Angiography as Reference Standard. AJR Am. J. Roentgenol..

[19] Kim Y., Choi A.D., Telluri A., Lipkin I., Bradley A.J., Sidahmed A., Jonas R., Andreini D., Bathina R., Baggiano A. (2023). Atherosclerosis Imaging Quantitative Computed Tomography (AI-QCT) to guide referral to invasive coronary angiography in the randomized controlled CONSERVE trial. Clin. Cardiol..

[20] Nurmohamed N.S., Cole J.H., Budoff M., Karlsberg R.P., Gupta H., Sullenberger L.E., Quesada C.G., Rahban H., Woods K.M., Uzzilia J.R. (2024). Impact of Atherosclerosis Imaging-Quantitative Computed Tomography on Diagnostic Certainty, Downstream Testing, Coronary Revascularization and Medical Therapy: The CERTAIN Study. Eur. Heart J. Cardiovasc. Imaging.

[21] Nakanishi R., Motoyama S., Leipsic J., Budoff M.J. (2019). How accurate is atherosclerosis imaging by coronary computed tomography angiography?. J. Cardiovasc. Comput. Tomogr..

[22] Muthulakshmi M., Kavitha G. (2019). Deep CNN with LM learning based myocardial ischemia detection in cardiac magnetic resonance images. Annu. Int. Conf. IEEE Eng. Med. Biol. Soc..

[23] Chen Z., Lalande A., Salomon M., Decourselle T., Pommier T., Qayyum A., Shi J., Perrot G., Couturier R. (2022). Automatic deep learning-based myocardial infarction segmentation from delayed enhancement MRI. Comput. Med. Imaging Graph..

[24] Ghanbari F., Joyce T., Lorenzoni V., Guaricci A.I., Pavon A.G., Fusini L., Andreini D., Rabbat M.G., Aquaro G.D., Abete R. (2023). AI Cardiac MRI Scar Analysis Aids Prediction of Major Arrhythmic Events in the Multicenter DERIVATE Registry. Radiology.

[25] Yildiz M., Ashokprabhu N., Shewale A., Pico M., Henry T.D., Quesada O. (2022). Myocardial infarction with non-obstructive coronary arteries (MINOCA). Front. Cardiovasc. Med..

[26] Herzog B.A., Husmann L., Valenta I., Gaemperli O., Siegrist P.T., Tay F.M., Burkhard N., Wyss C.A., Kaufmann P.A. (2009). Long-term prognostic value of 13N-ammonia myocardial perfusion positron emission tomography added value of coronary flow reserve. J. Am. Coll. Cardiol..

[27] Ziadi M.C., Dekemp R.A., Williams K.A., Guo A., Chow B.J., Renaud J.M., Ruddy T.D., Sarveswaran N., Tee R.E., Beanlands R.S. (2011). Impaired myocardial flow reserve on rubidium-82 positron emission tomography imaging predicts adverse outcomes in patients assessed for myocardial ischemia. J. Am. Coll. Cardiol..

[28] Juarez-Orozco L.E., Knol R.J.J., Sanchez-Catasus C.A., Martinez-Manzanera O., van der Zant F.M., Knuuti J. (2020). Machine learning in the integration of simple variables for identifying patients with myocardial ischemia. J. Nucl. Cardiol..

[29] Tonino P.A., De Bruyne B., Pijls N.H., Siebert U., Ikeno F., van’ t Veer M., Klauss V., Manoharan G., Engstrom T., Oldroyd K.G. (2009). Fractional flow reserve versus angiography for guiding percutaneous coronary intervention. N. Engl. J. Med..

[30] Yang F., Shi K., Chen Y., Yin Y., Zhao Y., Zhang T. (2023). Effect of 320-Row Computed Tomography Acquisition Technology on Coronary Computed Tomography Angiography-Derived Fractional Flow Reserve Based on Machine Learning: Systolic and Diastolic Scan Acquisition. J. Comput. Assist. Tomogr..

[31] Qiao H.Y., Tang C.X., Schoepf U.J., Tesche C., Bayer R.R., Giovagnoli D.A., Todd Hudson H., Zhou C.S., Yan J., Lu M.J. (2020). Impact of machine learning-based coronary computed tomography angiography fractional flow reserve on treatment decisions and clinical outcomes in patients with suspected coronary artery disease. Eur. Radiol..

[32] Xue J., Li J., Sun D., Sheng L., Gong Y., Wang D., Zhang S., Zou Y., Shi J., Xu W. (2022). Functional Evaluation of Intermediate Coronary Lesions with Integrated Computed Tomography Angiography and Invasive Angiography in Patients with Stable Coronary Artery Disease. J. Transl. Int. Med..

[33] Schuessler M., Saner F., Al-Rashid F., Schlosser T. (2022). Diagnostic accuracy of coronary computed tomography angiography-derived fractional flow reserve (CT-FFR) in patients before liver transplantation using CT-FFR machine learning algorithm. Eur. Radiol..

[34] Halvorsen S., Mehilli J., Cassese S., Hall T.S., Abdelhamid M., Barbato E., De Hert S., de Laval I., Geisler T., Hinterbuchner L. (2022). 2022 ESC Guidelines on cardiovascular assessment and management of patients undergoing non-cardiac surgery. Eur. Heart J..

[35] Lee J.M., Choi G., Koo B.K., Hwang D., Park J., Zhang J., Kim K.J., Tong Y., Kim H.J., Grady L. (2019). Identification of High-Risk Plaques Destined to Cause Acute Coronary Syndrome Using Coronary Computed Tomographic Angiography and Computational Fluid Dynamics. JACC Cardiovasc. Imaging.

[36] Hong H., Li C., Gutierrez-Chico J.L., Wang Z., Huang J., Chu M., Kubo T., Chen L., Wijns W., Tu S. (2022). Radial wall strain: A novel angiographic measure of plaque composition and vulnerability. EuroIntervention.

[37] Virmani R., Kolodgie F.D., Burke A.P., Farb A., Schwartz S.M. (2000). Lessons from sudden coronary death: A comprehensive morphological classification scheme for atherosclerotic lesions. Arter. Thromb. Vasc. Biol..

[38] Puchner S.B., Liu T., Mayrhofer T., Truong Q.A., Lee H., Fleg J.L., Nagurney J.T., Udelson J.E., Hoffmann U., Ferencik M. (2014). High-risk plaque detected on coronary CT angiography predicts acute coronary syndromes independent of significant stenosis in acute chest pain: Results from the ROMICAT-II trial. J. Am. Coll. Cardiol..

[39] Pu J., Mintz G.S., Biro S., Lee J.B., Sum S.T., Madden S.P., Burke A.P., Zhang P., He B., Goldstein J.A. (2014). Insights into echo-attenuated plaques, echolucent plaques, and plaques with spotty calcification: Novel findings from comparisons among intravascular ultrasound, near-infrared spectroscopy, and pathological histology in 2,294 human coronary artery segments. J. Am. Coll. Cardiol..

[40] Park S.J., Ahn J.M., Kang D.Y., Yun S.C., Ahn Y.K., Kim W.J., Nam C.W., Jeong J.O., Chae I.H., Shiomi H. (2024). Preventive percutaneous coronary intervention versus optimal medical therapy alone for the treatment of vulnerable atherosclerotic coronary plaques (PREVENT): A multicentre, open-label, randomised controlled trial. Lancet.

[41] Narula J., Stuckey T.D., Nakazawa G., Ahmadi A., Matsumura M., Petersen K., Mirza S., Ng N., Mullen S., Schaap M. (2024). Prospective deep learning-based quantitative assessment of coronary plaque by computed tomography angiography compared with intravascular ultrasound: The REVEALPLAQUE study. Eur. Heart J. Cardiovasc. Imaging.

[42] Rinehart S., Raible S.J., Ng N., Mullen S., Huey W., Rogers C., Pursnani A. (2024). Utility of Artificial Intelligence Plaque Quantification: Results of the DECODE Study. J. Soc. Cardiovasc. Angiogr. Interv..

[43] Omori H., Matsuo H., Fujimoto S., Sobue Y., Nozaki Y., Nakazawa G., Takahashi K., Osawa K., Okubo R., Kaneko U. (2023). Determination of lipid-rich plaques by artificial intelligence-enabled quantitative computed tomography using near-infrared spectroscopy as reference. Atherosclerosis.

[44] Gu J., Sanchez R., Chauhan A., Fazio S., Wong N. (2022). Lipid treatment status and goal attainment among patients with atherosclerotic cardiovascular disease in the United States: A 2019 update. Am. J. Prev. Cardiol..

[45] Zelias A., Bednarek A., Dykla D., Wysocka R., Szczygiel P., Dudek D. (2024). Lipid goal achievement one month after myocardial infarction: Observational real-world study from Polish population. Kardiol. Pol..

[46] Lin A., Manral N., McElhinney P., Killekar A., Matsumoto H., Kwiecinski J., Pieszko K., Razipour A., Grodecki K., Park C. (2022). Deep learning-enabled coronary CT angiography for plaque and stenosis quantification and cardiac risk prediction: An international multicentre study. Lancet Digit. Health.

[47] Masuda T., Nakaura T., Funama Y., Okimoto T., Sato T., Higaki T., Noda N., Imada N., Baba Y., Awai K. (2019). Machine-learning integration of CT histogram analysis to evaluate the composition of atherosclerotic plaques: Validation with IB-IVUS. J. Cardiovasc. Comput. Tomogr..

[48] Gerbaud E., Weisz G., Tanaka A., Kashiwagi M., Shimizu T., Wang L., Souza C., Bouma B.E., Suter M.J., Shishkov M. (2016). Multi-laboratory inter-institute reproducibility study of IVOCT and IVUS assessments using published consensus document definitions. Eur. Heart J. Cardiovasc. Imaging.

[49] Blanco P.J., Ziemer P.G.P., Bulant C.A., Ueki Y., Bass R., Raber L., Lemos P.A., Garcia-Garcia H.M. (2022). Fully automated lumen and vessel contour segmentation in intravascular ultrasound datasets. Med. Image Anal..

[50] Yang J., Faraji M., Basu A. (2019). Robust segmentation of arterial walls in intravascular ultrasound images using Dual Path U-Net. Ultrasonics.

[51] Abdolmanafi A., Duong L., Dahdah N., Adib I.R., Cheriet F. (2018). Characterization of coronary artery pathological formations from OCT imaging using deep learning. Biomed. Opt. Express.

[52] Zhao X., Wang Y., Chen R., Li J., Zhou J., Liu C., Zhou P., Sheng Z., Chen Y., Song L. (2021). Prognostic value of characteristics of plaque combined with residual syntax score among patients with STEMI undergoing primary PCI: An intravascular optical coherence tomography study. Thromb. J..

[53] Avital Y., Madar A., Arnon S., Koifman E. (2021). Identification of coronary calcifications in optical coherence tomography imaging using deep learning. Sci. Rep..

[54] Gharaibeh Y., Prabhu D., Kolluru C., Lee J., Zimin V., Bezerra H., Wilson D. (2019). Coronary calcification segmentation in intravascular OCT images using deep learning: Application to calcification scoring. J Med Imaging (Bellingham).

[55] Barbato E., Gallinoro E., Abdel-Wahab M., Andreini D., Carrie D., Di Mario C., Dudek D., Escaned J., Fajadet J., Guagliumi G. (2023). Management strategies for heavily calcified coronary stenoses: An EAPCI clinical consensus statement in collaboration with the EURO4C-PCR group. Eur. Heart J..

[56] Park S., Araki M., Nakajima A., Lee H., Fuster V., Ye J.C., Jang I.K. (2022). Enhanced Diagnosis of Plaque Erosion by Deep Learning in Patients With Acute Coronary Syndromes. JACC Cardiovasc. Interv..

[57] Jia H., Dai J., Hou J., Xing L., Ma L., Liu H., Xu M., Yao Y., Hu S., Yamamoto E. (2017). Effective anti-thrombotic therapy without stenting: Intravascular optical coherence tomography-based management in plaque erosion (the EROSION study). Eur. Heart J..

[58] Yin D., Mintz G.S., Song L., Chen Z., Lee T., Kirtane A.J., Parikh M.A., Moses J.W., Fall K.N., Jeremias A. (2020). In-stent restenosis characteristics and repeat stenting underexpansion: Insights from optical coherence tomography. EuroIntervention.

[59] Shinohara H., Kodera S., Ninomiya K., Nakamoto M., Katsushika S., Saito A., Minatsuki S., Kikuchi H., Kiyosue A., Higashikuni Y. (2021). Automatic detection of vessel structure by deep learning using intravascular ultrasound images of the coronary arteries. PLoS ONE.

[60] Abdel-Wahab M., Richardt G., Joachim Buttner H., Toelg R., Geist V., Meinertz T., Schofer J., King L., Neumann F.J., Khattab A.A. (2013). High-speed rotational atherectomy before paclitaxel-eluting stent implantation in complex calcified coronary lesions: The randomized ROTAXUS (Rotational Atherectomy Prior to Taxus Stent Treatment for Complex Native Coronary Artery Disease) trial. JACC Cardiovasc. Interv..

[61] Zhang L., Wahle A., Chen Z., Lopez J.J., Kovarnik T., Sonka M. (2018). Predicting Locations of High-Risk Plaques in Coronary Arteries in Patients Receiving Statin Therapy. IEEE Trans. Med. Imaging.

[62] Mach F., Baigent C., Catapano A.L., Koskinas K.C., Casula M., Badimon L., Chapman M.J., De Backer G.G., Delgado V., Ference B.A. (2020). 2019 ESC/EAS Guidelines for the management of dyslipidaemias: Lipid modification to reduce cardiovascular risk. Eur. Heart J..

[63] Garcia-Garcia H.M., Waksman R., Melaku G.D., Garg M., Beyene S., Wlodarczak A., Kerai A., Levine M.B., van der Schaaf R.J., Torzewski J. (2023). Temporal Changes in Coronary Plaque as Assessed by an Artificial Intelligence Based Optical Coherence Tomography: From the First-in-Human Trial on DREAMS 3G Scaffold. Eur. Heart J. Cardiovasc. Imaging.

[64] Lee J., Kim J.N., Gharaibeh Y., Zimin V.N., Dallan L.A.P., Pereira G.T.R., Vergara-Martel A., Kolluru C., Hoori A., Bezerra H.G. (2023). OCTOPUS—Optical coherence tomography plaque and stent analysis software. Heliyon.

[65] Follmer B., Williams M.C., Dey D., Arbab-Zadeh A., Maurovich-Horvat P., Volleberg R., Rueckert D., Schnabel J.A., Newby D.E., Dweck M.R. (2023). Roadmap on the use of artificial intelligence for imaging of vulnerable atherosclerotic plaque in coronary arteries. Nat. Rev. Cardiol..

[66] Guo X., Maehara A., Matsumura M., Wang L., Zheng J., Samady H., Mintz G.S., Giddens D.P., Tang D. (2021). Predicting plaque vulnerability change using intravascular ultrasound + optical coherence tomography image-based fluid-structure interaction models and machine learning methods with patient follow-up data: A feasibility study. Biomed. Eng. Online.

[67] Min H.S., Ryu D., Kang S.J., Lee J.G., Yoo J.H., Cho H., Kang D.Y., Lee P.H., Ahn J.M., Park D.W. (2021). Prediction of Coronary Stent Underexpansion by Pre-Procedural Intravascular Ultrasound-Based Deep Learning. JACC Cardiovasc. Interv..

[68] Dangas G.D., Caixeta A., Mehran R., Parise H., Lansky A.J., Cristea E., Brodie B.R., Witzenbichler B., Guagliumi G., Peruga J.Z. (2011). Frequency and predictors of stent thrombosis after percutaneous coronary intervention in acute myocardial infarction. Circulation.

[69] Thondapu V., Bourantas C.V., Foin N., Jang I.K., Serruys P.W., Barlis P. (2017). Biomechanical stress in coronary atherosclerosis: Emerging insights from computational modelling. Eur. Heart J..

[70] Motwani M., Dey D., Berman D.S., Germano G., Achenbach S., Al-Mallah M.H., Andreini D., Budoff M.J., Cademartiri F., Callister T.Q. (2017). Machine learning for prediction of all-cause mortality in patients with suspected coronary artery disease: A 5-year multicentre prospective registry analysis. Eur. Heart J..

[71] Antonopoulos A.S., Sanna F., Sabharwal N., Thomas S., Oikonomou E.K., Herdman L., Margaritis M., Shirodaria C., Kampoli A.M., Akoumianakis I. (2017). Detecting human coronary inflammation by imaging perivascular fat. Sci. Transl. Med..

[72] Oikonomou E.K., Williams M.C., Kotanidis C.P., Desai M.Y., Marwan M., Antonopoulos A.S., Thomas K.E., Thomas S., Akoumianakis I., Fan L.M. (2019). A novel machine learning-derived radiotranscriptomic signature of perivascular fat improves cardiac risk prediction using coronary CT angiography. Eur. Heart J..

[73] Oikonomou E.K., Antonopoulos A.S., Schottlander D., Marwan M., Mathers C., Tomlins P., Siddique M., Kluner L.V., Shirodaria C., Mavrogiannis M.C. (2021). Standardized measurement of coronary inflammation using cardiovascular computed tomography: Integration in clinical care as a prognostic medical device. Cardiovasc. Res..

[74] Hoshino M., Zhang J., Sugiyama T., Yang S., Kanaji Y., Hamaya R., Yamaguchi M., Hada M., Misawa T., Usui E. (2021). Prognostic value of pericoronary inflammation and unsupervised machine-learning-defined phenotypic clustering of CT angiographic findings. Int. J. Cardiol..

[75] Lin A., Kolossvary M., Yuvaraj J., Cadet S., McElhinney P.A., Jiang C., Nerlekar N., Nicholls S.J., Slomka P.J., Maurovich-Horvat P. (2020). Myocardial Infarction Associates With a Distinct Pericoronary Adipose Tissue Radiomic Phenotype: A Prospective Case-Control Study. JACC Cardiovasc. Imaging.

[76] Nurmohamed N.S., Min J.K., Anthopolos R., Reynolds H.R., Earls J.P., Crabtree T., Mancini G.B.J., Leipsic J., Budoff M.J., Hague C.J. (2024). Atherosclerosis quantification and cardiovascular risk: The ISCHEMIA trial. Eur. Heart J..

[77] Betancur J., Otaki Y., Motwani M., Fish M.B., Lemley M., Dey D., Gransar H., Tamarappoo B., Germano G., Sharir T. (2018). Prognostic Value of Combined Clinical and Myocardial Perfusion Imaging Data Using Machine Learning. JACC Cardiovasc. Imaging.

[78] Shaw L.J., Iskandrian A.E. (2004). Prognostic value of gated myocardial perfusion SPECT. J. Nucl. Cardiol..

[79] Kwiecinski J., Tzolos E., Meah M.N., Cadet S., Adamson P.D., Grodecki K., Joshi N.V., Moss A.J., Williams M.C., van Beek E.J.R. (2022). Machine Learning with (18)F-Sodium Fluoride PET and Quantitative Plaque Analysis on CT Angiography for the Future Risk of Myocardial Infarction. J. Nucl. Med..

[80] Haro Alonso D., Wernick M.N., Yang Y., Germano G., Berman D.S., Slomka P. (2019). Prediction of cardiac death after adenosine myocardial perfusion SPECT based on machine learning. J. Nucl. Cardiol..

[81] Ulloa Cerna A.E., Jing L., Good C.W., vanMaanen D.P., Raghunath S., Suever J.D., Nevius C.D., Wehner G.J., Hartzel D.N., Leader J.B. (2021). Deep-learning-assisted analysis of echocardiographic videos improves predictions of all-cause mortality. Nat. Biomed. Eng..

[82] Hague D.C. (2019). Benefits, Pitfalls, and Potential Bias in Health Care AI. N. Carol. Med. J..

[83] Ledford H. (2019). Millions of black people affected by racial bias in health-care algorithms. Nature.

[84] Sanchez-Martinez S., Camara O., Piella G., Cikes M., Gonzalez-Ballester M.A., Miron M., Vellido A., Gomez E., Fraser A.G., Bijnens B. (2021). Machine Learning for Clinical Decision-Making: Challenges and Opportunities in Cardiovascular Imaging. Front. Cardiovasc. Med..

