# Supplementary material for: Artificial Intelligence in Imaging for Personalized Management of Coronary Artery Disease
Source: J Clin Med. 2025 Jan 13;14(2):462. doi: 10.3390/jcm14020462 (PMC11765647; doi:10.3390/jcm14020462)
Supplement: Supplementary file 1 [file jcm-14-00462-s001.zip › jcm-3361817-supplementary.pdf]

Supplementary Table S1. Currently ongoing trials of AI-based tools for the management of coronary artery disease
ICA – invasive coronary angiography, CCTA – coronary computed tomography angiography, IVUS – intravascular ultrasound, OCT – optical coherence tomography, ECHO – echocardiography, CMR – cardiac magnetic resonance, AI – artificial intelligence, STEMI - ST-Segment Elevation Myocardial Infarction, TVF – target vessel failure, FFR – fractional flow reserve, MACE – major adverse cardiovascular events, LVEF – left ventricular ejection fraction, CAD – coronary artery disease

| Title | Clinical Trial ID | Imaging | Aim | Primary endpoint | Estimated numer of participants | Type of the study | Main country |
|---|---|---|---|---|---|---|---|
| Artificial Intelligence With DEep Learning on COROnary Microvascular Disease (AIDECORO) | NCT04598997 | ICA | Identification of patients with poor prognosis criteria at the time of hospitalization for STEMI | Death or re-hospitalization for heart failure – predictive accurracy | 600 prospective, 1000 retrospective | Observational | France |
| Artificial Intelligence With Deep Learning and Genes on Cardiovascular Disease | NCT03877614 | All available | Analysis of the differences in the individualization of cardiovascular disease to predict patient's recovery and improve medical quality | The rate of myocardial infarction, stroke, death, cardiovascular death, heart failure with hospitalization in 5 years | 5000 | Observational | Taiwan |
| Artificial Intelligence for Preventing Heart Disease (AiPHD): Observational, Single Center, | NCT06029387 | CCTA | Identification of patients at high-risk of disease progression | Death by cardiovascular events or non-fatal myocardial infarction 36 months from CCTA | 2500 retrospective, 500 prospective | Observational | Italy |

| | | | | | | | |
|---|---|---|---|---|---|---|---|
| Prospective and Retrospective Study (AiPHD) | | | | | | | |
| Clinical Impact of Intravascular Ultrasound-Based Artificial Intelligence Technologies (INNOVATE-PCI) | NCT05807841 | IVUS | Validation of diagnostic performances and clinical impact of IVUS and coronary angiography based models | Culprit-related TVF 2 years after stent implantation | 3000 | Observational | South Korea |
| Identifying Vulnerable CoronAry PLaqUes With Artificial IntElligence-assisted CT Angiography (VALUE) | NCT06025305 | CCTA | Detection and quantification of coronary plaques from CCTA | Sensitivity and specificity of AI-assisted coronary CT angiography on identifying vulnerable plaques compared to intravascular imaging | 2000 | Observational | China |
| Automatic Anatomical and Functional Classification of Coronary Arteries With Artificial Intelligence. (COROSCAN-IA) | NCT05810610 | CCTA | Validation of CCTA based AI model in detection of stenosis requiring intervention | Predictive performance, at the coronary vessel level, of an intelligent Coronary CT AI based image analysis system on the detection of a stenosis requiring intervention, versus invasive coronary angiography with | 1670 | Observational | France |

| | | | | | | | |
|---|---|---|---|---|---|---|---|
| | | | | reference measurement (FFR). | | | |
| Risk Evaluation by COronary CTA and Artificial intelliGence Based fuNctIonal analyZing tEchniques - I (RECOGNIZE-I) | NCT05884008 | CCTA | Establishment of a coronary artery disease risk stratification system by CCTA and anatomic, functional and radiomic analysis, assisted by artificial intelligence | Coronary artery plaque risk level | 300 | Observational | China |
| The AIPLAQUE Study: An Artificial Intelligence-based Prospective Study to Analyze PLAQUE Using CCTA | NCT05750082 | CCTA | Construction of CCTA based AI model for identifying coronary plaque components | Accuracy of automated plaque characterization and functional significance of coronary stenosis using CCTA images computation | 100 | Observational | China |
| Risk Evaluation by COronary CTA and Artificial IntelliGence Based fuNctIonal analyZing tEchniques - II (RECOGNIZE-II) | NCT05856110 | CCTA | Evaluation of accuracy of early identification of cardiovascular high-risk groups | MACE – Cardiovascular death, Myocardial infarction, stroke in 2 years | 2000 | Observational | China |
| Identification of Neoatherosclerosis | NCT04220437 | ICA, OCT | Construction of the AI algorithm | Neoatherosclerosis | 90 | Observational | China |

| | | | | | | | |
|---|---|---|---|---|---|---|---|
| in ISR Patients Based on Artifical Intelligence | | | allowing for identification of neoatherosclerosis without the use of OCT | | | | |
| Warning Model of Myocardial Remodeling After Acute Myocardial Infarction Using Multimodal Feature Structure Technology | NCT06062316 | CMR | Estabilshment of the model allowing for early identification of myocardial remodelling after myocardial infarction | Quantitative characterization of myocardial remodeling, cardiac magnetic resonance imaging quantifying necrotic areas and recoverable myocardium within the edematous myocardium after myocardial infarction | 3000 | Observational | China |
| Evaluation on the Effectiveness and Safety of RuiXin-CoronaryAI for Diagnosis of Coronary Artery Stenosis | NCT05320185 | CCTA | Evaluation of RuiXin-CoronaryAI accuracy in diagnosis of coronary artery stenosis | Per-vessel diagnostic sensitivity and specificity of RuiXin-CoronaryAI for diagnosis of coronary artery stenosis | 615 | Observational | China |
| CCTA, CACS and ECG Stress Testing in Patients With Suspected CAD: Precision Phenotyping and | NCT04424121 | CCTA | Development of new risk stratification algorithms for patients with stable symptoms | MACE<br>Chain-pest rehospitalization<br>Both in 18 months | 900 | Randomized (1:1:1) | Greece |

| | | | | | | | |
|---|---|---|---|---|---|---|---|
| Financial Evaluation (DATASET) | | | and low to intermediate probability of CAD | | | | |
| Evaluating AI-Gatekeeper Software in Coronary Artery Stenosis Screening: A Multicenter RCT (AIGatekeeper) | NCT06178900 | X-ray and ECHO | Validation of efficiacy, safety, and cost-effectivenes of AI-gatekeeper in assisting clinicians for predicting coronary artery stenosis without CCTA and CAG | Composite of MACE and diagnosis without significant coronary artery stenosis (<50%) on coronary artery disease testing in 24 weeks | 450 | Randomized (1:1) | South Korea |
| Evaluation of a Free-breathing Cardiac Cine-MRI Sequence With Image Reconstructions by Deep-Learning in Ischemic Heart Disease (CINEDL) | NCT05105984 | CMR | Validation of DL algorithm for automatic reconstruction of cine-MRI | difference of LVEF measurements between Deep Learning reconstruction and the classic cine-CMR sequence | 75 | Observational | France |
| Peri-luminal COROnary CTa AI-driven radiOMICS to Identify Vulnerable Patients (CORO-CTAIOMICS) | NCT06029777 | CCTA | Developing of algorithm capable of predicting of MACE by automatic analysis of periluminal coronary tissue | All-cause mortality, myocardial infarction, due to unstable angina or heart hospitalization failure, late coronary revascularization in 48 months | 2190 | Observational | Italy |

| | | | | | | | |
|---|---|---|---|---|---|---|---|
| (CORO-CTAIOMICS) | | | radiomics from CCTA | | | | |
| Role of On-site CT-derived FFR in the Management of Suspect CAD Patients (TARGET) | NCT03901326 | CCTA | Evaluating the role of CT-FFR in ruling out patients without significant CAD compared to regular diagnostic pathway | Rate of ICA without obstructive CAD or intervention in 90 days | 1216 | Randomized (1:1) | China |
